# Improving the Performance of Temperature Index Snowmelt Model of SWAT by Using MODIS Land Surface Temperature Data

**DOI:** 10.1155/2014/823424

**Published:** 2014-08-03

**Authors:** Yan Yang, Takeo Onishi, Ken Hiramatsu

**Affiliations:** ^1^United Graduate School of Agricultural Science, Gifu University, 1-1 Yanagido, Gifu 501-1193, Japan; ^2^Faculty of Applied Biological Sciences, Gifu University, 1-1 Yanagido, Gifu 501-1193, Japan

## Abstract

Simulation results of the widely used temperature index snowmelt model are greatly influenced by input air temperature data. Spatially sparse air temperature data remain the main factor inducing uncertainties and errors in that model, which limits its applications. Thus, to solve this problem, we created new air temperature data using linear regression relationships that can be formulated based on MODIS land surface temperature data. The Soil Water Assessment Tool model, which includes an improved temperature index snowmelt module, was chosen to test the newly created data. By evaluating simulation performance for daily snowmelt in three test basins of the Amur River, performance of the newly created data was assessed. The coefficient of determination (*R* ^2^) and Nash-Sutcliffe efficiency (NSE) were used for evaluation. The results indicate that MODIS land surface temperature data can be used as a new source for air temperature data creation. This will improve snow simulation using the temperature index model in an area with sparse air temperature observations.

## 1. Introduction

In most of the middle and high latitude regions, snow accumulation and subsequent snowmelt are considered as the most important hydrological processes, because the stream hydrograph is dominated by spring snowmelt [1]. In addition, nutrient transport from land to sea is significantly influenced by spring flood processes [2, 3]. Hence, knowledge of the spring snowmelt process is essential not only for hydrological modeling, but also for further study of nutrient dynamics and transport in middle and high latitude regions. Distributed hydrological models have been proven useful and applicable to investigate streamflow and nutrient transport in snowmelt-dominated basins [4, 5].

Recently, the physically based energy-balance method has been demonstrated to be accurate and powerful for calculating snowmelt processes [6–8]. However, the demand for accurate and variable input data and complex parameterization still limits applicability of the method [9, 10]. Conversely, the temperature index (henceforth, *T*-*I*) method has been widely used despite its simplicity for the following reasons [11]: (1) wide availability of air temperature data, (2) relative ease of air temperature interpolation and forecasting, and (3) computational simplicity. Thus, hydrological models such as the Soil Water Assessment Tool [12], Hydrological Simulation Program Fortran (HSPF, [13]), MIKE [14], and Snowmelt Runoff Model (SRM, [15]) have adopted the *T*-*I* method to simulate snow accumulation and the snowmelt process. Because *T*-*I* is based on an assumption that the relationship between ablation and air temperature is usually expressed in the form of positive temperature sums [11], the air temperatures are obviously one of the most important variables for this method.

Air temperature (*T*_*a*_) data can be easily obtained in regions where meteorological observation network is dense, but many remote areas have sparse stations. However, with development of earth observations, MODIS remotely sensed land surface temperature (LST) data have proven powerful for creating *T*_*a*_ data. For example, Kloog et al. [16] successfully applied the spatial smoothing method to evaluate daily air temperature data using MODIS LST data in Massachusetts, United States. Zhu et al. [17] also used MODIS LST to evaluate daily and subdaily maximum and minimum air temperature on the northern Tibetan Plateau. Zakšek and Schroedter-Homscheidt [18] reviewed that there are three different methods commonly applied for estimating the *T*_*a*_ based on the LST data: (1) the statistical methods; (2) the temperature-vegetation index methods (TVX); (3) energy-balance methods. They reported that the statistical methods generally perform well, within the spatial and time frame they were derived from, but require large amounts of data to train the algorithms [19]. The TVX method is based on the assumption that, for an infinitely thick canopy, the top-of-canopy temperature is the same as within the canopy [17] and uses the Normalized Difference Vegetation Index (NDVI) as a key input variable. However, the assumption of linear and negative slope between LST and NDVI is not always applicable and is influenced by seasonality, ecosystem type, and soil moisture variability [19, 20], and the period of created data is limited by the periods of both LST and NDVI data. In addition, Zhu et al. [17] also found that the results of the statistical method are similar to the TVX method. Although the energy-balance methods are physically based, the major disadvantage of this method is the requirement of large amounts of information often not provided by remote sensing [19]. The linear relationship between MODIS LST and *T*_*a*_ data has been demonstrated in different study regions [21–24]. Thus, the linear regression method is a common choice for *T*_*a*_ data estimation using MODIS LST data. Colombi et al. [25] used the linear regression method and MODIS LST data to generate average daily temperature in Italian alpine areas, and they proved that the result of the linear regression method was superior to that of the spatial interpolation method. Shen and Leptoukh [26] also used the linear regression relationship between air temperature and MODIS LST data to create new daily air temperature data in northern China and central Russia.

One critical disadvantage of using MODIS LST data is that the period of newly created air temperature data is limited by the operational period of the satellite. However, we frequently need historical data, especially for long-term hydrological simulations. Thus, it is necessary to find an easy and effective way to create spatially dense and temporally long-termair temperature data. Motivated by this unsolved problem, we set our research objectives as follows: (1) development of a simple method to create accurate air temperature data over a longer period; (2) hydrological simulation of the snowmelt process using newly created air temperaturedata for three test basins in the Amur River basin; and (3) evaluation of the validity of the newly created air temperature data by analysis of simulated results.

## 2. Study Area and Data

### 2.1. Test Basins

**Figure 1:**
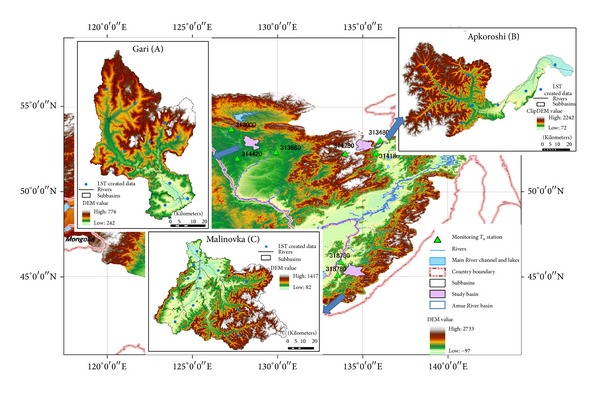
Study area and location of three test basins: Gari (A), Apkoroshi (B), and Malinovka (C).

Three basins were selected for model testing, which are located in the upper, middle, and lower stream of the Amur River basin. Basic geographic characteristics of the basins are shown in Figure 1 and Table 1. The Amur River is the tenth longest in the world and is recognized as an important dissolved iron source for the Sea of Okhotsk [27]. There are four distinct phases in the Amur water regime: spring floods, summer low water, summer and autumn floods, and winter low water. The main water source is rainfall, supplying 70–80% of total water, and snowmelt during spring floods adds 10–20% [28]. In the upper stream (basin A, Gari), the annual temperature is −2.4°C and the annual precipitation is 494 mm, and in the lower stream (basin B, Apkoroshi), the annual temperature is −0.1°C and the annual precipitation is 641 mm. In the middle stream (basin C, Malinovka), the mean annual temperature is 1.1°C and the annual precipitation is 593 mm [29].

### 2.2. Data for Hydrological Simulation

Detailed structure of the snow melt component of SWAT model is explained in the following sections. To generate input data needed for SWAT, a digital elevation model (DEM), soil data, land use and land cover (LULC) data, and weather data are required [30]. In addition, discharge data are required for calibration of hydrological simulations.

A Shuttle Radar Topographic Mission (SRTM 90 m) DEM was used to delineate subbasins of the test basins. We applied the same flow accumulation threshold (200 km^2^) in watershed delineation of all three test basins. The subbasins were delineated by the ArcSWAT interface. As shown in Figure 1, there are nine subbasins in basin A and 11 in basin B and basin C. The land use/land cover map was constructed by combined use of vegetation maps of China, Mongolia, and Russia and satellite images [31]. Soil data were taken from the Harmonized World Soil Database (HWSD, [32]), obtained from the International Institute for Applied Systems Analysis (IIASA). The spatial resolution of LULC data is as same as DEM data (90 m), and the resolution of soil data is 1 km.

Temporal resolution of all weather data was daily. Asian Precipitation Highly Resolved Observational Data Integration towards Evaluation of Water Resources (APHRODITE, [33]) was used for daily precipitation data. The most important driving data of distributed hydrological models are of accurate precipitation. It has been shown that APHRODITE can give good performance in this study area [34, 35]. Maximum air temperature (*T*_*a*,max⁡_), minimum air temperature (*T*_*a*,min⁡_), and wind speed data were obtained from the Global Historical Climatic Network-Daily (GHCN-Daily, [36]) of the National Climate Data Center (NCDC). The SWAT model calculates the distance between geometer center of subbasin and the candidate weather station to attach the nearest station for each subbasin as the unique driving station. In our study, the SWAT model selected eight monitoring air temperature stations (Figure 1 and Table 2) for SWAT model. Relative humidity and solar radiation data were from the NCEP-DOE reanalysis 2 dataset [37] on the website of the NOAA Earth System Research Laboratory. Because we focused on improving snowmelt simulations in the *T*-*I* method, daily monitoring runoff data during March through May were used for the calibration period to obtain optimized parameter values. The years 1983–1987 were selected for basins B and C, and because of lack of the monitoring, 1983, 1984, 1986, and 1988 were used for basin A. Runoff data were provided by the Russian Federal Service for Hydrometeorology and Environmental Monitoring (Roshydromet).

### 2.3. MODIS Land Surface Temperature Data

The AQUA/MODIS daily LST data at 1 km spatial resolution of both daytime and nighttime were acquired from mid-2002 to 2010. (https://lpdaac.usgs.gov/products/modis_products_table/myd11a1). For different MODIS LST data (TERRA/MODIS and AQUA/MODIS), Mostovoy et al. [23] already proved that the AQUA and TERRA have no significant difference in *T*_*a*_ estimation. In addition, Vancutsem et al. [20] also proved that the AQUA data can give reasonable results even if its observation period is shorter than TERRA. MODIS LST data are derived from thermal infrared bands 31 (10.78–11.28 *μ*m) and 32 (11.77–12.27 *μ*m). Atmospheric effects are corrected by a generalized split-window algorithm [38]. The latest LST data are version 005; these datasets have error less than 1°C within the range −10 to 50°C, assuming that surface emissivity is known [19, 39]. In addition, ground-based validation has shown that errors were less than 1°C at homogeneous surfaces such as water, crop, and grassland [39].

## 3. Methodology

### 3.1. Long-Term Air Temperature Data Creation and Validation

**Figure 2:**
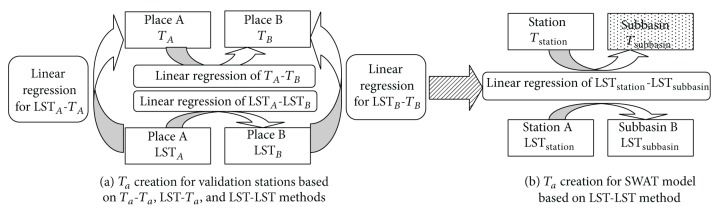
Diagram of *T*_*a*_ creation methods based on *T*_*a*_ and LST data for station validation and SWAT model.

The simplest method to estimate the air temperature is to create a linear regression equation between *T*_*a*_ at points *A* and *B* (Figure 2) using observed *T*_*a*_ data. In this case, the linear regression equation can be written as follows:

(1) $$\begin{array}{c} T_{a,B}=a_{1}\times T_{a,A}+b_{1}. \end{array}$$

Here, *a*_1_ and *b*_1_ are coefficients of the linear regression equation, and subscripts *A* and *B* indicate the points.

We call this the *T*_*a*_-*T*_*a*_ method. If monitoring data at both points *A* and *B* are available, we can use the method. However, if we have no data at a point we need to know, the method is not applicable. Here, we use the monitoring *T*_*a*_ stations and their nearest stations (Table 2) to evaluate the linear relationships between two different places (Figure 2).

We used three indices: coefficient of determination (*R* ^2^), mean absolute error (MAE), and root mean square error (RMSE) for the evaluation. Equations for *R* ^2^, MAE, and RMSE are as follows:

(2) $$\begin{array}{c} R^{2}=\left\{\frac{\sum_{i=1}^{n}\left(y_{i}-\overset{-}{y}\right)\left({\hat{y}}_{i}-\overset{-}{{\hat{y}}_{i}}\right)}{\left[\sum_{i=1}^{n}{\left(y_{i}-\overset{-}{y}\right)}^{2}\right]\left[\sum_{i=1}^{n}{\left({\hat{y}}_{i}-\overset{-}{{\hat{y}}_{i}}\right)}^{2}\right]}\right\},\\ \text{MAE}=\frac{1}{n}\overset{n}{\underset{i=1}{\sum }}\left|y_{i}-{\hat{y}}_{i}\right|,\\ \text{RMSE}={\left(\frac{\sum_{i=1}^{n}{\left|y_{i}-{\hat{y}}_{i}\right|}^{2}}{n}\right)}^{1/2}. \end{array}$$

Here, *y*_*i*_ is observed *T*_*a*_ on day *i*, ${\hat{y}}_{i}$ is created air temperature on day *i* by each method, $\overset{-}{y}$ is the average value of observed *T*_*a*_, $\overset{-}{{\hat{y}}_{i}}$ is the average value of created air temperature, and *n* is the total number of days. The results of linear correlation analysis for *T*_*a*_ are shown in Table 3 and discussed in Section 4.1.

Further, the study of Sun et al. [24] presented a theoretical derivation of linear regression relationships between *T*_*a*_ and LST and proved that the *T*_*a*_ can be mainly explained by the LST in the linear regression equation, and they also showed that the errors of created *T*_*a*_ based on the linear regression method are limited in a reasonable range, in the North China Plain. In addition, Mostovoy et al. [23] also proposed the similar method to estimate *T*_*a*_ at any point from LST at that point. By constructing linear regression equations between *T*_*a*_ and LST at 161 monitoring station points in the state of Mississippi, they also found a common relationship between LST and *T*_*a*_, irrespective of location. Furthermore, both of these researches indicate that the first order coefficient of linear equation between *T*_*a*_ and LST is equal to 1. Here, we use the monitoring *T*_*a*_ data and LST data to estimate the air temperature between them; we call this the LST-*T*_*a*_ method. In this case, the linear regression equation can be written as

(3) $$\begin{array}{c} T_{a}=\text{LST}+\text{const}. \end{array}$$

We also validate the linear regression relationships between the *T*_*a*_ and LST in all stations (Table 4); the *R* ^2^ is over 0.95 for both daily maximum and minimum *T*_*a*_-LST analysis for all station pairs. In addition, the results also showed that the first order coefficient (slope or “*a*”) of linear regression equations (Table 4) is very close to 1. These results also correspond to the theoretical analysis of previous researches in China North Plain [24] and Mississippi of USA [23].

However, the results of *T*_*a*_-LST indicate that this method extends the errors compared with the *T*_*a*_-*T*_*a*_ method. Furthermore, a disadvantage of this method is that because LST data are necessary for this method; it can only be applied to periods after MODIS was launched. As already addressed, we frequently need historical air temperature data to execute hydrological models that include snow accumulation and snowmelt processes. Moreover, many watersheds have very sparse observed air temperature data. Because both the *T*_*a*_-*T*_*a*_ and LST-*T*_*a*_ methods are unsuitable for such common cases, we developed a new method as follows.

In the first step, a linear regression equation of LST between two points is created as follows:

(4) $$\begin{array}{c} {\text{LST}}_{B}^{\prime }=a_{2}\times {\text{LST}}_{A}+b_{2}. \end{array}$$

Here, *a*_2_ and *b*_2_ are coefficients of the linear regression equation, and subscripts *A* and *B* indicate the points. LST_*B*_′ is the result of the predicted LST value of point B from (4).

We conducted the linear regression analysis of LST data in all station pairs. According to the results (Table 5), the same as the previous two methods, it is clear that high linear correlations are obtained in all station pairs for LST data.

In addition, based on the linear analysis results of *T*_*a*_-LST of station pairs, and using the *T*_*a*_-LST method, we can estimate *T*_*a*,*A*_ and *T*_*a*,*B*_ at the same time:

(5) $$\begin{array}{c} T_{a,A}≅{\text{LST}}_{A}+{\text{const}}_{A}, \end{array}$$

(6) $$\begin{array}{c} T_{a,B}≅{\text{LST}}_{B}+{\text{const}}_{B}. \end{array}$$

Based on the results of linear correlation analysis of LST data (Table 5), we neglect the error between the LST_*B*_′ and LST_*B*_ and by substituting LST_*B*_ in (6) by LST_*A*_′, *T*_*a*,*B*_ can be expressed by LST_*A*_ as

(7) $$\begin{array}{c} T_{a,B}=a_{2}\times {\text{LST}}_{A}+b_{2}+{\text{const}}_{B}. \end{array}$$

Combining (7) with (5), we get

(8) $$\begin{array}{c} T_{a,B}=a_{2}\times T_{a,A}+b_{2}+{\text{const}}_{B}-a_{2}\times {\text{const}}_{A}. \end{array}$$

Because the *T*_*a*_-LST relationship in the study basins can only be acquired in the point which monitors both LST and *T*_*a*_ data, const_*B*_ cannot be obtained as *T*_*a*_ is not monitored in place B. In this study, we take the const_*B*_ − *a*_2_ × const_*A*_ as an entirety. It is clear that if this entirety can be ignored, the equation will be simplified drastically.

Thus, we calculated the const_*B*_ − *a*_2_ × const_*A*_ in all station pairs based on the results listed in Tables 4 and 5. The results (Table 6) indicate that in station pair 4-4*N* there is a relative large error compared with other station pairs for both daily maximum and minimum analysis. The error is larger in the daily minimum analysis of 2-2*N* and 6-6*N*, and it is also larger in the 7-7*N* for the daily maximum analysis. In addition, the average value of all station pairs is 0.38°C for daily maximum analysis and 0.45°C for daily minimum analysis. However, we also recognize that the effects of *T*_*a*_ data on the snowmelt processes are based not only on the “point” or “situ” scale but also on its accurate distribution in the entire basin. Thus, considering relative small average errors (Table 6), we ignore the const_*B*_ − *a*_2_ × const_*A*_ of (8) and the new equation is

(9) $$\begin{array}{c} T_{a,B}=a_{2}\times T_{a,A}+b_{2}.\; \end{array}$$

This means that once we acquire coefficients *a*_2_ and *b*_2_ from linear regression analysis of LST, we can estimate *T*_*a*,*B*_ using a known *T*_*a*,*A*_. We call this the LST-LST method. We performed a linear regression analysis for creation of *T*_*a*_ based on both daily maximum and minimum LST data. However, according to the limited monitoring period of MODIS LST data, the linear regression equations are formulated only in the entire period. We compared estimated *T*_*a*_ from the LST-LST method with the *T*_*a*_-*T*_*a*_ method, using the same station pairs listed in Table 2, and also the errors caused from the approximation are discussed, especially the approximations of (8) which may cause more errors for the *T*_*a*_ estimation. The comparison was for both the entire period and spring snowmelt period. We defined the snowmelt period as March through May. The results are presented in Section 4.1.

### 3.2. Air Temperature Calculation in the SWAT Model

The SWAT model can consider orographic effects on air temperature by dividing the subbasin into multiple elevation bands [40]. This significantly influences snow cover and snowmelt processes. The equation is

(10) $$\begin{array}{c} T_{\text{band}}=T_{a}+\left({EL}_{\text{band}}-{EL}_{\text{gage}}\right)\times \frac{TLAPS}{1000}. \end{array}$$

Here, *T*_band_ is calculated *T*_*a*_ in each elevation band (°C), *T*_*a*_ is temperature recorded at a monitoring gage (°C), *EL*_band_ is mean elevation of each elevation band (m), *EL*_gage_ is elevation of an existing monitoring gage (m), *TLAPS* is temperature lapse rate (°C/km), and 1000 is a unit conversion factor from meters to kilometers. By multiplying *T*_band_ and areal percentage of a band and summing over all elevation bands, we can obtain average *T*_*a*_ of each subbasin. All test basins were divided into ten elevation bands, based on an equal interval of elevation.

### 3.3. Snowmelt Simulation in the SWAT Model

Based on the basic concept of the *T*-*I* method, the snowmelt module of the SWAT model is as follows (time interval is daily):

(11) $$\begin{array}{c} {\text{SNO}}_{\text{mlt}}=b_{\text{mlt}}\times {\text{SNO}}_{\mathrm{cov}}\times \left(\frac{T_{\text{snow}}+T_{\text{mx}}}{2}-T_{\text{mlt}}\right). \end{array}$$

Here, SNO_mlt_ is the amount of snowmelt (mm H_2_O), *b*_mlt_ is the melt factor (mm H_2_O/day-°C), and SNO_cov⁡_ is the fraction of the hydrological response unit (HRU) area covered by snow. The HRU is divided by using land use and soil types in each subbasin. The HRU is the simulated unit in the SWAT model; the simulation of water budget in SWAT is based on the HRU. *T*_snow_ is snow pack temperature (°C), *T*_mx_ is maximum *T*_*a*_ (°C), and *T*_mlt_ is the base temperature above which snowmelt is allowed (°C). *b*_mlt_ allows seasonal variation with maximum and minimum values occurring on the summer and winter solstices. SNO_cov⁡_ allows nonuniform snow cover caused by factors such as shading, land cover, and topography.

Consider

(12) $$\begin{array}{c} T_{\text{snow}(d_{n})}=T_{\text{snow}(d_{n}-1)}\times l_{\text{sno}}+{\overset{-}{T}}_{\text{av}}\times \left({1-l}_{\text{sno}}\right). \end{array}$$

Here, *T*_snow(*d*_*n*_)_ is snow pack temperature on a given day (°C), *T*_snow(*d*_*n*_−1)_ is snow pack temperature on the previous day (°C), *l*_sno_ is snow temperature lag, and ${\overset{-}{T}}_{\text{av}}$ is mean *T*_*a*_ on a given day. In SWAT model, the ${\overset{-}{T}}_{\text{av}}$ is the arithmetic mean value of daily maximum *T*_*a*_ and minimum *T*_*a*_.

### 3.4. Model Calibration and Evaluation

To evaluate different input *T*_*a*_ datasets influences on SWAT model snowmelt simulation, we conducted hydrological simulations under two different scenarios: (1) Elev*T*_*a*_, which used the original *T*_*a*_ data with elevation band method; (2) New*T*_*a*_, which used the newly created *T*_*a*_ data based on the LST-LST method without elevation bands. For other settings such as watershed delineations, HRU generations, input data (except *T*_*a*_), and initial range of calibration parameters, we keep them the same for all different scenarios in each test basin.

Model parameters were calibrated using the Sequential Uncertainty Fitting Version 2 (SUFI-2) method [30].  Table 7 lists the calibration parameters for each scenario. *TLAPS* is only included in the Elev*T*_*a*_ scenario. Two different parameter sets were constructed for testing the performance of each scenario. One is the so-called “the least parameters setting” and the other is “complete parameters setting.”

For the least parameters setting, to test the performance of two different *T*_*a*_ datasets, we fixed the values of parameters that might significantly influence snowmelt simulations; that is, *SNOCOV*50 = 0.5, *SNOCOV* *M* *X* = 300, *SFTMP* = 1°C, *SMTMP* = 0.5°C, *SMFMX* = 4.5 mm/H_2_O-°C, *SFMN* = 4.5 mm/H_2_O-°C, and *TIMP* = 0°C. Remaining parameters were calibrated to control the entire simulation hydrograph. Parameters listed in Table 7 were all applied to evaluate the influence of all factors on snowmelt simulations with the complete parameters setting.

Objective functions used for parameter optimization in runoff simulations were the Nash-Sutcliffe efficiency (NSE) and coefficient of determination (*R* ^2^). The sensitivity of parameters was evaluated by the *t*-test of each parameter in the linear multiple regression, which was created using the parameters as arguments and the objective value (NSE) as the dependent variable. The NSE is calculated as follows:

(13) $$\begin{array}{c} \text{NSE}=1-\frac{\sum_{i=1}^{n}{\left(P_{i}-O_{i}\right)}^{2}}{\sum_{i=1}^{n}{\left(O_{i}-\overset{-}{O}\right)}^{2}}. \end{array}$$

Here, *O*_*i*_ is observed runoff on day *i*,$\;\overset{-}{O}$ is average observed runoff for the entire period, *P*_*i*_ is simulated runoff on day *i*, and *n* is the total number of days.

## 4. Results and Discussion

### 4.1. Comparison between the LST-LST and *T*_*a*_-*T*_*a*_ Methods

**Figure 3:**
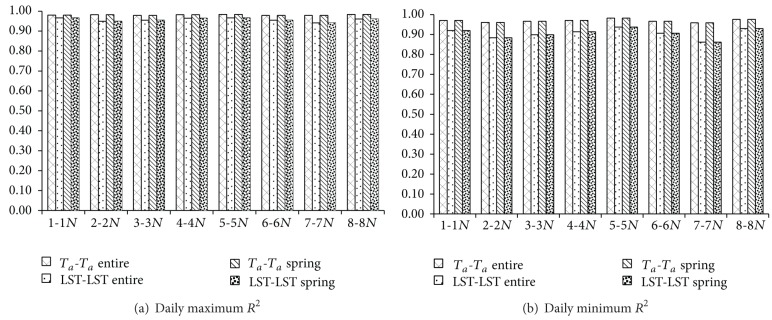
*R* ^2^ values of daily maximum and minimum *T*_*a*_ creation results for *T*_*a*_-*T*_*a*_ and LST-LST methods. In each group of bars, from left to right are *T*_*a*_-*T*_*a*_ (entire period), LST-LST (entire period), *T*_*a*_-*T*_*a*_ (spring), and LST-LST (spring).

**Figure 4:**
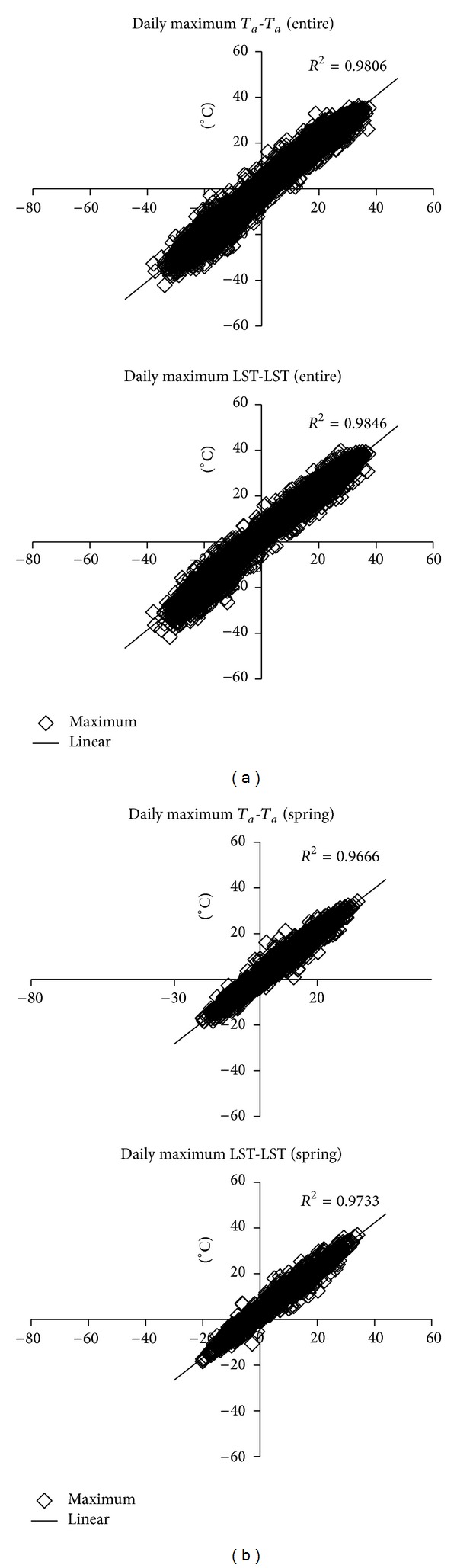
Scatter plot of daily maximum *T*_*a*_ creation results for *T*_*a*_-*T*_*a*_ and LST-LST methods during the entire period (a) and spring (b), taking station pair 1-1*N* as example.

**Figure 5:**
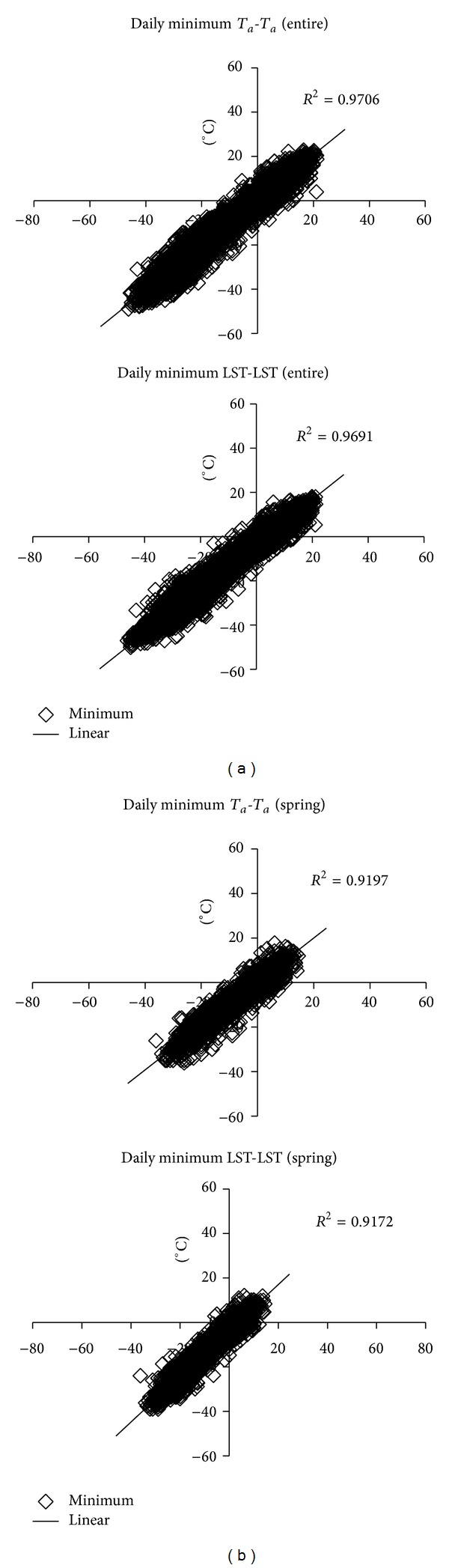
Scatter plot of daily minimum *T*_*a*_ creation results for *T*_*a*_-*T*_*a*_ and LST-LST methods during the entire period (a) and spring (b), taking station pair 1-1*N* as example.

**Figure 6:**
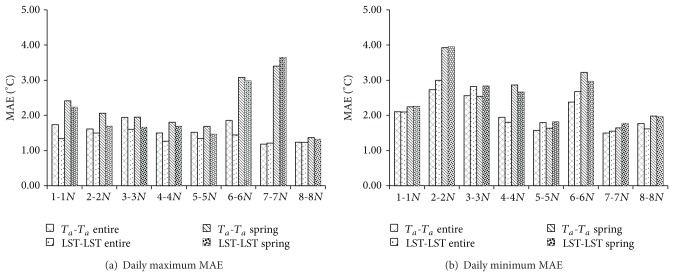
MAE values of daily maximum and minimum *T*_*a*_ creation results for *T*_*a*_-*T*_*a*_ and LST-LST methods. In each group of bars, from left to right are *T*_*a*_-*T*_*a*_ (entire period), LST-LST (entire period), *T*_*a*_-*T*_*a*_ (spring), and LST-LST (spring).

**Figure 7:**
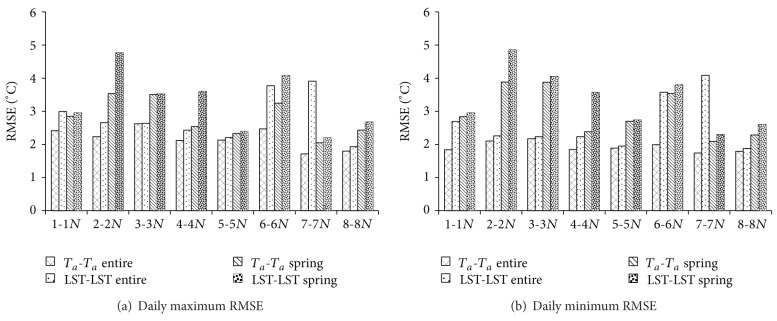
RMSE values of daily maximum and minimum *T*_*a*_ creation results for *T*_*a*_-*T*_*a*_ and LST-LST methods. In each group of bars, from left to right are *T*_*a*_-*T*_*a*_ (entire period), LST-LST (entire period), *T*_*a*_-*T*_*a*_ (spring), and LST-LST (spring).

The *R* ^2^ of the entire (year) period was over 0.95 for both maximum and minimum *T*_*a*_ estimation of *T*_*a*_-*T*_*a*_ method (Figures 3, 4, and 5). The results clearly demonstrate that based on the long monitoring period and abundant monitoring data there are high linear correlations in all station pairs. For the LST-LST method, except the station pairs 2-2*N* and 7-7*N*, all other station pairs are over 0.90. Although these *R* ^2^ were less than 0.95 in spring using both methods, they exceeded 0.9 in the *T*_*a*_-*T*_*a*_ method. Also, the results of the *R* ^2^ in spring period for LST-LST method showed the same trend as the entire period. For both the entire year and spring, MAE of the LST-LST method was slightly larger than that of the *T*_*a*_-*T*_*a*_ method (Figure 6). Overall, MAE performances were around 1.0−2.0°C in the entire year for daily maximum *T*_*a*_ prediction, but this increasedto 2.0−3.5°C in spring. For daily minimum *T*_*a*_ prediction, MAE was 1.5−2.5°C for the entire year and 1.5−4.0°C in the spring. The results of RMSE (Figure 7) show the same trend as MAE; RSME of the LST−LST method was larger than that of the *T*_*a*_-*T*_*a*_ method. The larger errors are obtained especially at the station pairs 2-2*N*, 4-4*N*, 6-6*N*, and 7-7*N*. These results highly agree with the results listed in Section 3.1 and Table 6, in which the potential errors caused by neglecting the item const_*B*_ − *a*_2_ × const_*A*_ of (8) are presented. Benali et al. [19] and Vancutsem et al. [20] demonstrated that the land cover may influence the *T*_*a*_ estimation results from statistical method based on MODIS LST data. Here, in our study, the station pairs 2-2*N*, 4-4*N*, 6-6*N*, and 7-7*N* all contained the land cover type wetland (2*N*, 4*N*, 6, and 7 station), and the land cover type of their corresponding stations is forest for station 2, shrub for stations 4 and 6*N*, and agriculture land for 7*N*. Further, Westermann et al. [41] presented that MODIS LST data exhibit the high difference between dry area and wet area during snowmelt season in the tundra of Svalbard, Norway. Hachem et al. [42] also concluded that the stagnant surface water which can modify the heat exchanges between the ground surface and the atmosphere leads to the difference between the air temperature and LST in Alaska. Obviously, the water content and vegetation cover are significantly different between the wetland and other land covers. Thus, in our study, the difference of water content in different land cover type is also the probable reason that induces the relative larger error in the station pairs: 2-2*N*, 4-4*N*, 6-6*N*, and 7-7*N*.

Furthermore, the statistical analysis method always needs abundant training data to generate stable results [18]. The amount of data listed in the Tables 3, 4,and 5 clearly suggested that the short observation period of the MODIS LST data might be an important factor that induces the larger errors in the LST-LST method. The results also indicate that during the spring, ranges of MAE and RMSE from both *T*_*a*_-*T*_*a*_ and LST-LST methods are increased compared with the entire year. Obviously, comparing with the entire period, the available data for the linear regression analysis is much less, and therefore this is the main reason that the *T*_*a*_-*T*_*a*_ method and the LST-LST method tend to include more error during the spring.

Although the errors are extended by the LST-LST method, the MAEs and RMSEs obtained herein were still within a reasonable range compared with earlier research in different regions. For example, based on the multistep linear regression method, Colombi et al. [25] predicted *T*_*a*_ in alpine areas of Italy. Their RMSE = 1.89°C for daily average temperature and maximum and minimum *T*_*a*_ were 2.47°C and 3.36°C, respectively, similar to our results. Shen et al. [26] used a single linear regression method to predict daily maximum and minimum *T*_*a*_ based on LST data, with error 2-3°C. Generally, although the error is enlarged in LST-LST method compared with the *T*_*a*_-*T*_*a*_ method, the various indexes still show that errors of the LST-LST method were within a reasonable range, and overall performance of the method demonstrated its capability for predicting daily maximum and minimum temperature.

### 4.2. Simulation Results of Elev*T*_*a*_ and New*T*_*a*_ Scenarios in the Least and Complete Parameters Settings

#### 4.2.1. Simulations with the Least Parameters Setting

**Figure 8:**
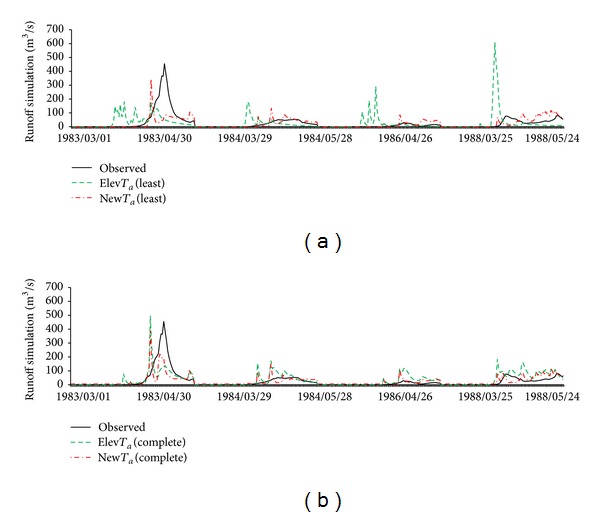
Spring snowmelt simulation results (March, April, and May) for the least parameters setting (a) and complete parameters setting (b) of basin A.

**Figure 9:**
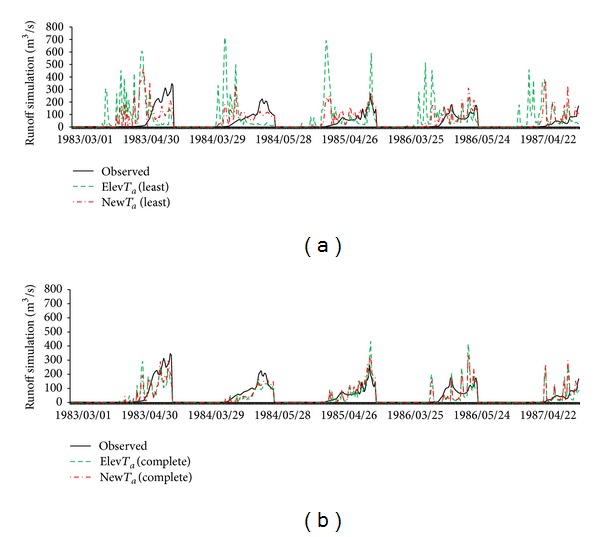
Spring snowmelt simulation (March, April, and May) results of the least parameters setting (a) and complete parameters setting (b) of basin B.

**Figure 10:**
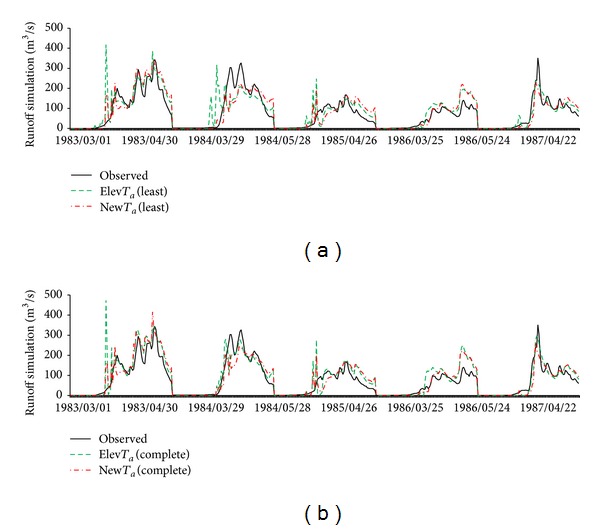
Spring snowmelt (March, April, and May) simulation results of least parameters setting (a) and complete parameters setting (b) of basin C.

Simulation results for bothscenarioswith the least parameters setting are shown in Figures 8, 9, and 10 and Table 8. Basically, the trend of these results in basin A was the same as in basin B. In basin A, the coefficient of determination of the Elev*T*_*a*_ and New*T*_*a*_ scenarios was 0.0 and 0.15, respectively, and NSE was −0.88 and 0.15. Snowmelt simulation results in both scenarios were overestimated in the early part of each year, while peak flow during snowmelt season was underestimated. In basin B, the coefficient of determination for Elev*T*_*a*_ and New*T*_*a*_ was 0.0 and 0.19. Although NSE values from both scenarios were negative and coefficients of determination were weak, New*T*_*a*_ gave better results than Elev*T*_*a*_, especially for NSE. Although all evaluation indices indicated poor performance, the results demonstrated that New*T*_*a*_ was superior to Elev*T*_*a*_. By contrast, for basin C, both scenarios achieved good results. There, the performance of New*T*_*a*_ (*R* ^2^ = 0.75, NSE = 0.73) was again better than Elev*T*_*a*_ (*R* ^2^ = 0.67, NSE = 0.63).

These results clearly show that performances of New*T*_*a*_ scenarios were always better than Elev*T*_*a*_ in all test basins. However, it was also suggested that the SWAT model cannot attain an acceptable level of simulation without model calibration. Although the test basins were in the same region, differences of snowmelt processes must still be considered. Because there were no additional observed data, parameter calibration is the only way to improve simulation performance to an acceptable level. Thus, simulation and parameter calibration with the complete parameters setting are discussed in the following section.

#### 4.2.2. Simulations with Complete Parameters Setting

(*1) Parameter Sensitivity*. Parameter sensitivity and optimal calibration values of the complete parameters setting are shown in Table 9. Obviously, parameters* SNOCOVMX* and* SNO50COV* that define the snow water content of the entire basin are in the top ranks for all test basins.* SMFMX* that affects daily snowmelt amount and* TIMP*, which strongly impacts the snow pack temperature, is also significant. Parameter* CN2*, which is generally recognized as the most important parameter for SWAT [43], was only significant in basin A and showed less sensitivity than* SNOCOVMX* or* SNO50COV*. Because our simulations only focused on the snowmelt period, it is reasonable that* CN2*, whose main function is to split net precipitation into surface flow and infiltration part, is not significant. One of the most influential parameters in the Elev*T*_*a*_ scenario is* TLAPS*, which is related to elevation change. The sensitivity of* TLAPS* was significant in basin B but insignificant in basin A and C. Considering the poor results of the least parameters setting, sensitivity results of the complete parameters setting strongly suggest that the parameters that influenced snowmelt varied between the test basins and that calibration is essential for the model to achieve better results. 

(*2) Simulation Results*. Simulation results for the complete parameters setting are shown in Figures 8, 9, and 10 and Table 8. In basin A, the coefficient of determination and NSE of the Elev*T*_*a*_ scenario (*R* ^2^ = 0.22, NSE = 0.11) were greatly improved in New*T*_*a*_ (*R* ^2^ = 0.42, NSE = 0.42). In basin B, the coefficient of determination and NSE of Elev*T*_*a*_ (*R* ^2^ = 0.43, NSE = 0.29) were improved in New*T*_*a*_, with respective increases of 0.16 and 0.20 (*R* ^2^ = 0.57, NSE = 0.49). For basin C, the NSE (*R* ^2^ = 0.79, NSE = 0.72) of Elev*T*_*a*_ was also improved slightly in New*T*_*a*_ (*R* ^2^ = 0.79, NSE = 0.75). Obviously, compared with the least parameters setting, results of the complete parameters setting were improved in all scenarios via parameter calibration. The results clearly show that, compared with the least parameters setting, results of the complete parameters setting can match the trend of monitoring data well. In basin A (1984, 1986, and 1987, Figure 8) and basin B (1983–1987, Figure 9) in particular, simulation results for the peak flow and early melt hydrograph can match monitoring data well, relative to the least parameters setting.

Aside from meteorological influences, land cover is another influence on snowmelt. Wetlands are believed to be a major factor in snowmelt simulation, because of its capacity for water storage [44]. Hydrological properties of these wetlands are very sensitive to variations of *T*_*a*_, seasonal precipitation, and other climatic factors [45]. To simulate snowmelt processes in wetlands, Fang et al. [46] applied a physically based approach to a wetland-dominated prairie basin in Canada. They found that the ability of wetlands to trap blowing snow in winter and store runoff water is a crucial feature of the hydrology, and this poses a substantial challenge to hydrological modeling. Wang et al. [47] and Yang et al. [48] demonstrated that the SWAT model's hydrological simulation component should be improved for wetland-dominated areas based on detailed wetland measurement data, such as annual water table depth and normal and maximum water storage capacities. Wetland covered over 25% of the entire basin A. However, in a data-sparse area, it is difficult to acquire enough data to conduct physically based snowmelt simulation of wetlands, and this is the likely reason that the NSE and *R* ^2^ were weak in that basin.

Furthermore, as shown in Table 1, the average slope of basin A is only 2.8 degrees. Obviously, snowmelt simulation in such a flat area is greatly influenced by input meteorological data, more than by topographic factors. The parameter sensitivity analysis results also demonstrate that elevation has less influence on snowmelt in this basin, based on the sensitivity rank of the* TLAPS* parameter (14th among the 16 parameters). However, the simulations indicate that the results are weak in both the least and calibrated Elev*T*_*a*_ scenarios and are greatly improved in the calibrated New*T*_*a*_ scenario.

SWAT model performance was more reasonable in basins B and C. A likely reason is that their elevation differences are significant; average slope of basin B is 18.2 degrees and that of basin C is 12.1 degrees (Table 1). Earlier studies proved that snowmelt performance of SWAT is reasonable in mountainous areas [40, 49, 50]. Furthermore, dominant land use types in the two basins are forest and pasture; some studies have demonstrated that SWAT model performance is stable in such basins. For example, Zhang et al. [43] used SWAT for the headwaters of the Yellow River, where land cover is dominated by pasture land. They found that SWAT could simulate snowmelt well, based on comparing the temperature index model with two other physical process-based models. Further, Debele et al. [51] demonstrated that SWAT performance was more stable than a physically based snowmelt model in a forest-dominated, watershed-scale basin. Although performance of the Elev*T*_*a*_ scenario was superior in the two basins relative to basin A, the New*T*_*a*_ data achieves better results.

In addition, based on the land cover information listed in the Tables 1 and 2, it is clear that the land cover type of the basin A is same as the *T*_*a*_ monitoring stations which are used for the LST-LST method. In the basin B, the shrub, forest, and wetland are land cover types of *T*_*a*_ monitoring stations. Although the land cover of the basin B (Table 1) showed that the pasture is also one of main land cover types the *T*_*a*_ monitoring station is consistent with the other land cover types (forest, shrub, and wetland; 79%). It is likely that the increment of NSE in the basin A and basin B can be explained by the homogeneity of land cover between monitoring *T*_*a*_ stations and test basins. Contrarily, wetland is the only land cover type of *T*_*a*_ stations and forest is the dominated land cover in the basin C. Consequently, the increment of NSE in this test basin is insignificant (Table 8). Hence, the results of our study indicate that the consistency between the land cover types of test basins and the *T*_*a*_ stations used for creation of new *T*_*a*_ can influence the increment of snowmelt simulation results. However, we should also recognize that the temperature is not the only factor that influences the snowmelt processes; the precipitation, topography, and wind speed also have great influences on the snowmelt. As previously mentioned (Section 3.4), we fixed other factors to make the *T*_*a*_ the only influence factor for the snowmelt simulation in each parameter settings; thus, the simulation results of New*T*_*a*_ scenarios can still prove the advantages of new created data for improving snow simulation in this study area. In addition, these results also suggest that the errors caused from the assumptions and approximations in the Section 3.1 have less effect on the final simulation results. The new created data performed well compared with the original *T*_*a*_ data.

Furthermore, although satisfactory results of runoff simulations are obtained with the Elev*T*_*a*_ scenario in basins B and C, calibrated* TLAPS* values indicated probable errors for the original data and method. Even though actual* TLAPS* values cannot be evaluated because of sparse monitoring of *T*_*a*_ data in our study area, its calibrated values (−8.8°C/km for basin A, −8.5°C/km for basin B, and −9.5°C/km for basin C) deviated strongly from the typical temperature lapse rate measured around the world (−6°C/km or −6.5°C/km). Our calibration results also clearly show that the lapse rate varied between test basins. This also applies for the basins (B and C) with large sensitivity differences, and the calibration values of the* TLAPS* also varied sharply.

These results strongly demonstrate that the elevation band method, which depended only on a fixed temperature lapse rate and the original sparse monitoring *T*_*a*_ dataset, had a less effect on daily snowmelt simulation in the study area. The results also clearly prove that the new high spatial density *T*_*a*_ data with no additional parameter or elevation modification can achieve superior results for both steep and flat basins.

## 5. Conclusions

The main objective of this study was to evaluate a new *T*_*a*_ data creation method to generate *T*_*a*_ data with high spatial density and accuracy, for improving the performance of snowmelt modeling using the *T*-*I* method. The research used a simple linear regression equation between two locations. The method was tested at eight pairs of air monitoring stations and compared with the regression method based on the monitoring data. Although the approximation of linear regression method might extend errors for *T*_*a*_ estimation, the results still demonstrate that this simple linear regression approach can create *T*_*a*_ data with limited errors range over long periods, and spatial density of the created data is very high. Snowmelt simulations with the newly created *T*_*a*_ data and original data were compared in three test basins with varying slope and land cover types. Both the least parameters and complete parameters settings were tested in all basins. The calibration results evaluated using different indices indicate that the newly created *T*_*a*_ data can obtain better simulation results than the original data and method, in all test basins. The simple linear regression using MODIS LST was generally successful and applicable in our study area. The research showed that using the newly created *T*_*a*_ data to improve the temperature index-based hydrological model (SWAT) is feasible, and the results of this new approach suggest that it could be a powerful means for extending the applicability of the *T*-*I* method to areas with sparse *T*_*a*_ data.

## Figures and Tables

**Table 1:** Basic geographic information of test basins.

Name	Gari (A)	Apkoroshi (B)	Malinovka (C)
Area (km^2^)	3315	4105	5006
Slope (degree)	2.8	18.3	12.1
Land-cover type composition (%)			
Forest	30	65	90
Wetland	25	7	0
Shrub	45	7	6
Farmland	0	0	4
Pasture	0	21	0

**Table 2:** Basic information of linear regression *T*_*a*_ stations.

ID	Station∗	Latitude	Longitude	Elevation (m)	LULC∗	NID	N_Station∗	Latitude	Longitude	Elevation (m)	LULC	P_Code∗	Dis∗ (km)
1	313000	53.700	127.300	229	Shrub	1N	312950	53.467	125.817	370	Shrub	1-1N	101.39
2	313480	53.050	136.033	153	Forest	2N	314160	52.417	136.500	73	Wetland	2-2N	77.24
3	313880	52.350	129.917	208	Wetland	3N	314590	51.350	130.433	261	Shrub	3-3N	116.79
4	314180	52.283	135.800	201	Shrub	4N	314160	52.417	136.500	73	Wetland	4-4N	49.95
5	314420	51.983	127.650	281	Forest	5N	314450	51.450	128.116	197	Agriculture	5-5N	67.47
6	314780	52.267	133.983	902	Wetland	6N	314740	51.633	133.267	384	Forest	6-6N	86.02
7	318730	45.867	133.733	101	Wetland	7N	509830	45.767	132.967	103	Agriculture	7-7N	61.30
8	318780	45.083	133.533	98	Wetland	8N	318730	45.867	133.733	101	Wetland	8-8N	87.38

*Station is the WMO code of stations used for validation of new created *T*_*a*_ data and SWAT model; N*_*Station shows the WMO code of the nearest station for each station in the “Station” column; P_code is the station pair name, LULC is the land cover type, and Dis is distance between the two stations.

**Table 3:** Results of daily maximum and minimum *T*_*a*_ linear regression analysis in each station pair.

Daily maximum *T*_*a*_-*T*_*a*_							Daily minimum *T*_*a*_-*T*_*a*_					
Sta_Pairs∗	DataNum∗	*a* ∗	*b* ∗	*R* ^2^	MAE (°C)	RMSE (°C)	DataNum∗	*a* ∗	*b* ∗	*R* ^2^	MAE (°C)	RMSE (°C)
1-1N	14328	1.01	0.16	0.98	1.74	2.41	14321	1.02	0.33	0.97	2.11	2.85
2-2N	13851	0.97	−0.31	0.98	1.61	2.23	13859	0.99	−4.07	0.96	2.73	3.53
3-3N	8640	1.02	−1.09	0.98	1.94	2.62	8641	1.01	1.61	0.97	2.56	3.51
4-4N	13980	0.96	0.22	0.98	1.50	2.12	13981	0.83	1.28	0.97	1.95	2.54
5-5N	13470	0.97	−0.14	0.98	1.52	2.13	13473	0.98	−0.83	0.98	1.57	2.33
6-6N	12851	0.93	−3.16	0.98	1.86	2.47	12849	0.95	−3.10	0.97	2.38	3.25
7-7N	13769	1.02	−0.33	0.99	1.19	1.71	13770	1.00	−0.99	0.98	1.50	2.05
8-8N	14537	0.98	1.03	0.99	1.24	1.80	14535	1.04	−0.06	0.98	1.77	2.43

Average		0.98	−0.45	0.98	1.58	2.19		0.98	−0.73	0.97	2.07	2.81

*Sta_Pairs is the station pair name, DataNum is the amount of data used for the regression analysis, “*a*” is the first order coefficient of linear regression equation, and the “*b*” is the interception of linear regression equation.

**Table 4:** Results of daily maximum and minimum *T*_*a*_-LST linear regression analysis in each station pair.

Daily maximum *T*_*a*_-LST							Daily minimum *T*_*a*_-LST					
ID	DataNum∗	*a* ∗	*b* ∗	*R* ^2^	MAE (°C)	RMSE (°C)	DataNum	*a*	*b*	*R* ^2^	MAE (°C)	RMSE (°C)
312950	954	1.13	−1.25	0.95	3.15	4.07	1439	1.05	−1.45	0.97	2.15	2.83
313000	1194	1.08	−2.29	0.95	2.86	3.81	1567	1.05	−2.39	0.97	2.21	2.92
313480	804	0.94	−3.18	0.94	1.95	2.69	1439	0.98	2.79	0.97	2.02	2.92
313880	1034	1.02	−2.33	0.95	2.64	3.50	1598	1.00	0.54	0.98	1.83	2.48
314160	1212	1.08	−3.37	0.97	2.38	3.20	1436	1.05	−0.51	0.98	2.19	2.97
314180	876	0.99	−1.43	0.95	2.10	2.85	1436	1.10	−2.77	0.97	2.16	3.03
314420	1135	1.08	−0.70	0.95	2.85	3.71	1592	1.01	−0.68	0.97	2.27	3.05
314450	1188	1.09	−0.84	0.95	3.03	4.06	1570	1.01	−0.24	0.97	2.35	3.11
314740	813	0.98	−2.68	0.93	2.24	2.97	1435	1.01	1.92	0.98	1.84	2.54
314780	720	1.14	−2.66	0.94	2.74	3.68	1460	1.02	−0.12	0.97	2.35	3.27
318730	1333	1.07	−1.15	0.97	2.31	3.20	1462	1.09	−1.78	0.96	2.38	3.18
318780	1286	1.06	−2.00	0.95	2.77	3.72	1224	1.11	−1.63	0.97	2.33	3.26
509830	1162	1.11	2.39	0.93	3.11	3.99	1449	1.02	−0.91	0.97	1.95	2.66

Average		1.06		0.95	2.63	3.50		1.04		0.97	2.16	2.94

*DataNum is the amount of data used for the regression analysis, “*a”* is the first order coefficient of linear regression equation, and the “*b”* is the interception of linear regression equation. (The *T*_*a*_ monitoring data of station 3*N* is till December 31, 1997, which cannot match the LST monitoring period*;* thus, there are no results for station 3*N*: 314590.)

**Table 5:** Results of daily maximum and minimum LST linear regression analysis in each station pair.

Daily maximum LST							Daily minimum LST					
Sta_Pairs∗	DataNum∗	*a* ∗	*b* ∗	*R* ^2^	MAE (°C)	RMSE (°C)	DataNum	*a*	*b*	*R* ^2^	MAE (°C)	RMSE (°C)
1-1N	826	0.95	−0.86	0.96	2.50	3.38	1305	1.00	−0.51	0.98	1.92	2.56
2-2N	644	0.90	−0.75	0.92	2.20	3.05	1182	0.91	−1.99	0.98	1.68	2.26
3-3N	742	1.01	−0.94	0.97	2.08	2.89	1323	1.03	1.96	0.98	1.77	2.32
4-4N	694	0.93	1.41	0.94	1.98	2.85	1192	0.88	−0.69	0.98	1.86	2.63
5-5N	978	0.94	0.22	0.97	2.21	2.98	1424	0.98	−1.36	0.99	1.36	1.80
6-6N	489	1.09	−2.80	0.90	2.70	3.75	1208	0.97	−5.25	0.97	2.28	2.98
7-7N	861	0.93	−2.85	0.93	3.09	3.89	1170	1.03	−1.69	0.97	1.85	2.51
8-8N	975	0.98	0.34	0.98	1.72	2.47	992	1.08	−0.81	0.97	2.32	3.04

Average		0.96	−0.78	0.94	2.31	3.16		0.98	−1.29	0.98	1.88	2.51

*Sta_Pairs is the station pair name, DataNum is the amount of data used for the regression analysis, “*a”* is the first order coefficient of linear regression equation, and the *b* is the interception of linear regression equation.

**Table 6:** Calculation results of the item const_B_ − *a*_2_ × const_A_ in (8).

Daily maximum LST-LST						Daily minimum LST-LST				
Sta_Pairs	const_A_ (°C)∗	*a*_2_*	const_A_ ∗ *a*_2_	const_B_ (°C)∗	const_B_ − const_A_ ∗ *a*_2_ (°C)	const_A_ (°C)	*a*_2_	const_A_ ∗ *a*_2_	const_B_ (°C)	const_B_ − const_A_ ∗ *a*_2_ (°C)
1-1N	−2.29	0.95	−2.17	−1.25	0.92	−2.39	1.00	−2.39	−1.45	0.94
2-2N	−3.18	0.90	−2.85	−3.37	−0.52	2.79	0.91	2.54	−0.51	−3.05
4-4N	−1.43	0.93	−1.33	−3.37	−2.04	−2.77	0.88	−2.43	−0.51	1.92
5-5N	−0.7	0.94	−0.66	−0.84	−0.18	−0.68	0.98	−0.67	−0.24	0.43
6-6N	−2.66	1.09	−2.89	−2.68	0.21	−0.12	0.97	−0.12	1.92	2.04
7-7N	−1.15	0.93	−1.07	2.39	3.46	−1.78	1.03	−1.83	−0.91	0.92
8-8N	−2	0.98	−1.96	−1.15	0.81	−1.63	1.08	−1.76	−1.78	−0.02

Average			−1.85	−1.47	0.38			−0.95	−0.50	0.45

*Sta_Pairs is the name of station pair, const_A_ is the interception value of linear regression equation in *T*_*a*_-LST method for point A (Table 4), *a*_2_ is the first order coefficient of linear regression equation of LST data linear regression results (Table 5), and const_B_ is the interception value of linear regression equation for *T*_*a*_-LST method in point B. (Because of the lack of *T*_*a*_-LST analysis result of station 3*N*, the station pair: 3-3*N* is excluded from the results.)

**Table 7:** Calibration parameters in Elev*T*_*a*_ and New*T*_*a*_ scenarios.

Parameters	Definition	Method	Range/percent
ALPHA_BF	Base-flow alpha factor (days)	V	0-1
CN2	Initial SCS CN II value	R	±50%
ESCO	Soil evaporation compensation factor	V	0.25–0.75
GW_DELAY	Groundwater delay (days)	V	0–30
SFTMP	Snowfall temperature (°C)	V	−5 to 5
SMTMP	Snowmelt base temperature (°C)	V	−5 to 5
SMFMN	Melt factor for snow on December 21 (mm H_2_O/°C-day)	V	0–3
SMFMX	Melt factor for snow on June 21 (mm H_2_O/°C-day)	V	3–9
SNO50COV	Fraction of snow volume represented by SNOCOVMX that corresponds to 50% snow cover	V	0.01–0.99
SNOCOVMX	Minimum snow water content corresponding to 100% snow cover (mm)	V	0–500
SOL_AWC	Average available water	R	±50%
SOL_K	Saturated conductivity	R	±50%
SURLAG	Surface runoff lag time (days)	V	1–24
TIMP	Snow pack temperature lag factor (°C)	V	0.01–1
TLAPS	Temperature lapse rate changed with the elevation (°C)	V	−10 to 0

Elev*T*_*a*_ scenario is elevation bands combined with the original monitoring air temperature data scenario, and New*T*_*a*_ scenario is the newly created air temperature data. V means the true value of the parameter and R means the relative range of the original parameter.

**Table 8:** Performance of Elev*T*_*a*_ and New*T*_*a*_ scenarios in the least parameters and complete parameters settings.

Basin	The least parameters settings				Complete parameters setting			
	Elev*T*_*a*_		New*T*_*a*_		Elev*T*_*a*_		New*T*_*a*_	
	*R* ^2^	NSE	*R* ^2^	NSE	*R* ^2^	NSE	*R* ^2^	NSE
A	0	−0.88	0.15	0.15	0.22	0.11	0.42	0.42
B	0	−3.98	0.19	−0.51	0.43	0.29	0.57	0.49
C	0.67	0.63	0.75	0.73	0.79	0.72	0.79	0.75

Elev*T*_*a*_ scenario is elevation bands combined with the original monitoring air temperature data scenario, and New*T*_*a*_ scenario is the newly created air temperature data. *R* ^2^ is coefficient of determination, and NSE is Nash-Sutcliffe efficiency.

**Table 9:** Complete parameters set calibration results, for both Elev*T*_*a*_ and New*T*_*a*_ scenarios.

Basin	A				B				C			
Parameter	Elev*T*_*a*_	Rank	New*T*_*a*_	Rank	Elev*T*_*a*_	Rank	New*T*_*a*_	Rank	Elev*T*_*a*_	Rank	New*T*_*a*_	Rank
ALPHA_BF	0.512	8	0.411	8	0.773	10	0.463	9	0.377	12	0.330	5
**CN2**	**0.062 **	**3**	**0.228**	**3**	**−0.184**	**4**	**−0.173**	**7**	**−0.390**	**10**	**−0.052**	**7**
ESCO	0.338	10	0.712	10	0.691	9	0.279	12	0.349	14	0.550	10
GW_DELAY	2.708	6	22.463	13	29.512	14	9.412	8	3.488	4	4.538	6
SFTMP	4.888	12	0.398	11	3.348	13	2.033	11	0.538	5	−2.438	13
SMFMN	0.574	5	1.183	9	0.704	11	1.595	6	0.520	9	1.342	8
**SMFMX**	**4.046 **	**7**	**7.607**	**2**	**8.486**	**2**	**3.764**	**4**	**4.211**	**6**	**6.002**	**3**
SMTMP	−2.283	13	2.538	7	4.728	12	−0.008	5	4.323	8	3.438	9
**SNO50COV**	**0.440 **	**1**	**0.820**	**4**	**0.644**	**3**	**0.608**	**14**	**0.728**	**1**	**0.644**	**2**
**SNOCOVMX**	**335.125 **	**2**	**320.375**	**1**	**465.875**	**1**	**464.625**	**1**	**257.375**	**3**	**275.625**	**1**
SOL_AWC	−0.219	11	0.225	12	0.323	15	−0.169	13	−0.165	7	0.414	12
SOL_K	0.043	15	0.148	14	0.304	7	0.455	10	0.487	15	0.353	11
SURLAG	1.017	4	1.420	6	1.776	6	17.163	3	18.969	11	1.615	4
TIMP	0.594	9	0.023	5	0.024	8	0.027	2	0.056	2	0.070	14
**TLAPS**	**−8.808 **	**14**			**−8.453**	**5**			**−9.518**	**13**		

Elev*T*_*a*_ scenario is elevation bands combined with the original monitoring air temperature data scenario, and New*T*_*a*_ scenario is the newly created air temperature data.
